# Endophytic Fungi: Biological Control and Induced Resistance to Phytopathogens and Abiotic Stresses

**DOI:** 10.3390/pathogens10050570

**Published:** 2021-05-08

**Authors:** Daniele Cristina Fontana, Samuel de Paula, Abel Galon Torres, Victor Hugo Moura de Souza, Sérgio Florentino Pascholati, Denise Schmidt, Durval Dourado Neto

**Affiliations:** 1 Department of Plant Production, Luiz de Queiroz College of Agriculture, University of São Paulo, Piracicaba 13418900, Brazil; daani_fontana@usp.br (D.C.F.); ddourado@usp.br (D.D.N.); 2 Plant Pathology Department, Luiz de Queiroz College of Agriculture, University of São Paulo, Piracicaba 13418900, Brazil; abel.torres@usp.br (A.G.T.); victorhugomour@gmail.com (V.H.M.d.S.); sfpascho@usp.br (S.F.P.); 3 Department of Agronomy and Environmental Science, Frederico Westphalen Campus, Federal University of Santa Maria, Frederico Westphalen 98400000, Brazil; denise@ufsm.br

**Keywords:** endophytes, resistance inducers, biological control, abiotic stress, plant-microbe interactions, sustainability, integrated pest management, microorganisms, plant disease control

## Abstract

Plant diseases cause losses of approximately 16% globally. Thus, management measures must be implemented to mitigate losses and guarantee food production. In addition to traditional management measures, induced resistance and biological control have gained ground in agriculture due to their enormous potential. Endophytic fungi internally colonize plant tissues and have the potential to act as control agents, such as biological agents or elicitors in the process of induced resistance and in attenuating abiotic stresses. In this review, we list the mode of action of this group of microorganisms which can act in controlling plant diseases and describe several examples in which endophytes were able to reduce the damage caused by pathogens and adverse conditions. This is due to their arsenal of molecules generated during the interaction by which they form a kind of biological shield in the plant. Furthermore, considering that endophytic fungi can be an important tool in managing for biotic and abiotic stresses due to the large amount of biologically active substances produced, bioprospecting this class of microorganisms is tending to increase and generate valuable products for agriculture.

## 1. Introduction

Agricultural production and global food security face substantial challenges. The world population is expected to exceed 9 billion by 2050, and an estimated 70% increase in food production over today’s production will be needed to ensure food security [1]. In this sense, the health of cultivated plants is of vital importance for the various economic sectors, because plants also provide essential products in addition to providing food for the population, such as wood, fibers, medicines, and bioenergy, among others. Plant diseases are responsible for quantitative and qualitative reduction in production, causing significant economic losses, and occasionally can lead to disastrous social consequences [2,3,4,5,6].

Plant diseases cause losses of up to 16% on a global scale [7], and studies have already pointed to losses directed at pathogens and more specifically to performed cultivations [3,8]. The potential for losses triggered by pathogens is indisputable, and their losses may vary depending on climatic factors, the culture and aggressiveness of the causal agent [8].

Diseases are traditionally managed through the use of genetic resistance (when available), and through the use of traditional chemical pesticides. The latter is highly used and has good efficiency in most cases [9]. However, this approach has experienced difficulties over the years due to its exhaustive use, which has led to the selection of pathogen populations which are resistant to the available active ingredients [10,11,12,13]. Driven by such a scenario, the study of complementary and alternative management measures has increased in recent years and has gained significant space in integrated management programs. As a result, biological control [14,15] and induced resistance [16,17,18,19] can be highlighted among the tools which have received attention. The potential of these two tools has been studied, explored and implemented in production fields, with numerous reports of successful cases in controlling pathogens.

**Figure 1:**
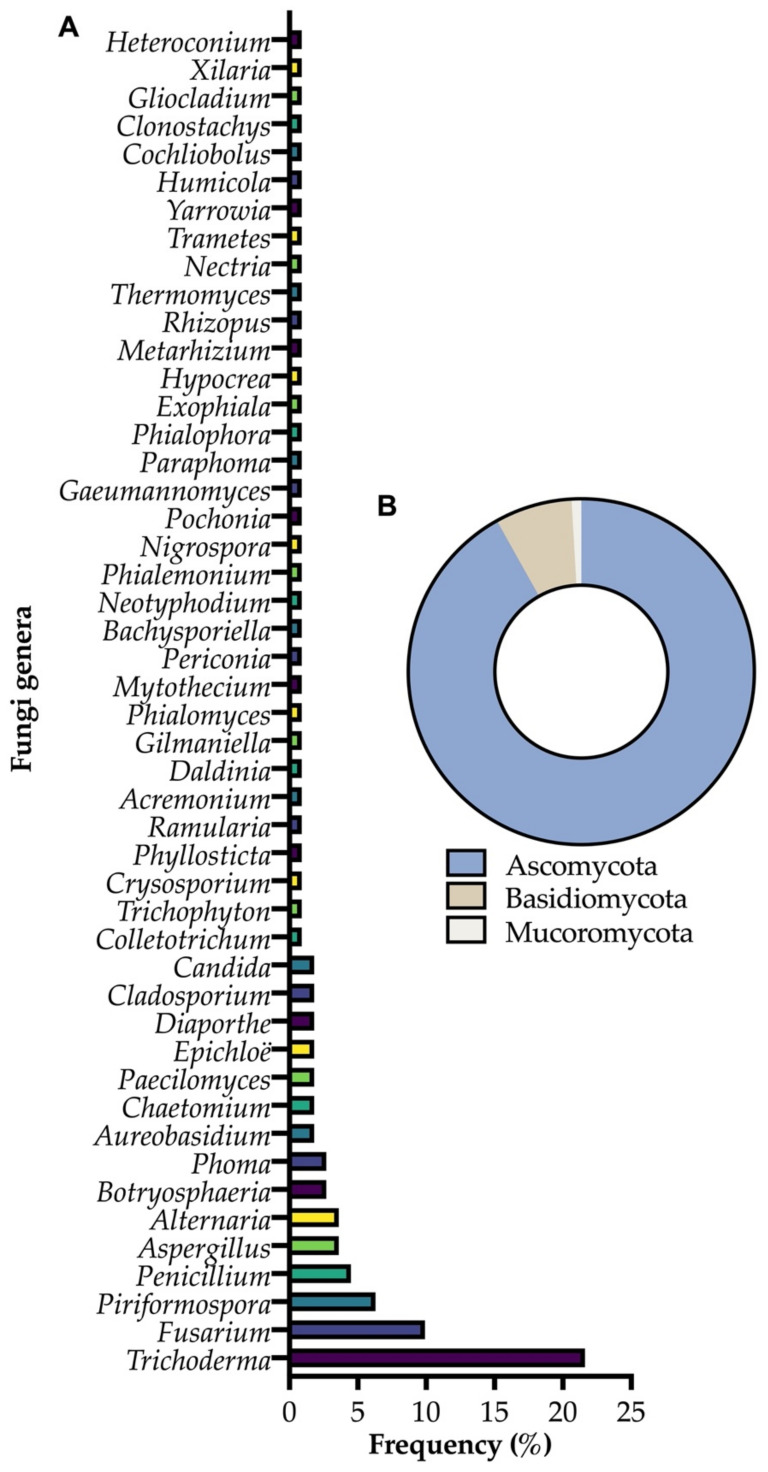
(**A**) Frequency of endophytic fungi genera cited in this review; (**B**) the phyla in which these microorganisms are classified.

Plants and microorganisms in nature live in interactions among them, which can affect plant growth, development and even defense responses to biotic and abiotic stresses [20]. Endophytic fungi are among the microorganisms that live in interaction with plants and can be used in biological control and induced resistance, and comprise one of the most interesting groups with high potential for use and high diversity (Figure 1). They present advantages since they internally colonize tissues and therefore remain protected from more hostile environmental conditions which could threaten their survival [21]. Thus, endophytic fungi are increasingly being studied due to their ability to assist in plant health. For example, regarding induced resistance, *Piriformospora indica* is able to induce resistance in *Musa* spp. against *Fusarium oxysporum* f. sp. *cubense* tropical race 4 by increasing the activities of antioxidant enzymes [22]. On the other hand, the endophytic fungus *Fusarium oxysporum* strain EF119 sensu lato acts as a biocontrol agent for tomato plants against oomycetes such as *Phytophthora infestans* [23].

Endophytes have received attention both as biological control agents and as activators of the plant’s defense response to biotic and abiotic stresses. Both approaches generally have satisfactory results and have the potential to be used as auxiliary strategies to traditional control and to be implemented in integrated disease management systems. The biological pesticide market in Brazil grew more than 70% in 2018, with a turnover of around US$ 127.2 million. This value exceeded the percentage presented by the international market, where the expectation was that the sector would earn US$5 billion in 2020 and reach US$11 billion in 2025 [24]. 

In this review, we discuss how endophytic fungi can benefit and act in plant protection. To do so, we approach three examples of phyla and several different genera within these, although there is a predominance of endophytic fungi such as *Trichoderma*, *Fusarium* and *Piriformospora* (Figure 1). The use of endophytic fungi as biological control agents and resistance inducers is detailed, emphasizing some of the most recent information on this topic which has been explored. In addition, the basis of biological control and stress-induced resistance is highlighted to facilitate understanding of applying endophytic fungi in this context, and in the context of integrated management. Finally, perspectives are presented to better understand how the endophytic fungi area should evolve in the coming years. Although endophytic bacteria can also act to protect plants against biotic [25,26,27] and abiotic stresses, this is not the focus of this review.

## 2. Endophytic Fungi

Endophytic microorganisms were first defined as those which live inside plant tissues, whether in asymptomatic infections (or not), and either in antagonistic or symbiotic interactions [28]. Later, any microorganisms which colonize the interior of aerial plant tissues in at least one stage of their life cycle without causing apparent damage to the host plant were considered endophytes [29]. After a few years of study, Azevedo and Araújo [30] defined endophytic microorganisms as all those cultivable (or not) which inhabit the interior of plant tissues, without causing damage to the host, and which do not develop visible external structures.

More recent views have been considered conceptualizing endophytic microorganisms as those that live in healthy plant tissues without causing obvious symptoms of infection in the host plant, and their existence is characterized as being abundant in nature [31]. The long-term coexistence of endophytes and host plants makes their relationship complex, so that endophytes can produce the same or similar active secondary metabolites as plants [31].

The colonization of plant tissues does not occur by chance, but probably because they were selected and adapted to grow in this niche. This is evident due to the energy used by the plant in producing biomass for the endophyte, being compensated by adaptive improvements resulting from the presence of the microorganisms [32]. The intense chemotactic signaling in the endophytic-host interaction also suggests that these microorganisms are not merely accidental opportunists, but are the result of a co-evolutionary adaptation between them [33].

Endophytes associated with plants represent an untapped source of new natural and bioactive products, with more than 20,000 described substances [34], of which 51% have new structures and 80% have biological activity [35]. For example, some have antimicrobial, antioxidant and anti-tumor activities [36,37,38,39]. This can be explained by the ecological theory, which establishes that this metabolic production is dependent on the ecological niche in which the microorganism is inserted and the consequent biotic and abiotic interactions [40].

Endophytic fungi inhabit a similar ecological niche to that occupied by phytopathogens, thus being able to protect their environment and control them through competition, production of antagonistic substances, direct parasitism or even inducing resistance or tolerance [41]. It is important to consider that some fungi which are endophytic for one plant species may be pathogenic for another species. In the same sense, the production of compounds such as antibiotics, for example, suggests that endophytes can control plant diseases [41]. 

Both hosts and endophytes are benefited in the interaction among them. On the one hand, the microorganism benefits from protection, nutrition and shelter in the plant. On the other hand, endophytes also help their hosts by stimulating their growth, development, adaptation, and stress tolerance [42,43]. Protection against diseases occurs by reducing the infection levels, as well as suppressing and reducing the growth of pathogens [44,45]. In this sense, it is suggested that the presence of endophytes during the evolutionary process allowed the plants to grow better and be more resistant to insects, herbivorous animals and pathogenic organisms. The same can be inferred regarding adverse environmental conditions such as low humidity and/or high temperatures [46].

The main focus in studying endophytic organisms is on the benefits promoted in the host plant’s health, in which they can “protect” plants against pests and pathogens, increasing growth, resistance to stress, and produce chemical compounds such as enzymes, alkaloids, hormones and antibiotics [47]. In turn, these compounds can present considerable toxicity, as is the case of the alkaloids produced by these fungi [33], which can help plants in the battle against pathogens.

The beneficial effect of the plant-endophytic association has received attention, and therefore these microorganisms have become an important tool in modern agriculture [47]. In addition, endophytic fungi can be genetically altered in order to introduce characteristics of interest in host plants [48].

The plants provide an environment in their interior for a high diversity of endophytic fungi. These microorganisms can colonize leaves, branches and roots, without causing damage to the hosts [47], systemically inhabiting the apoplast, vascular tissues and in some cases the cell interior [49]. For example, in cacao grown in Bahia State, Brazil, it was observed that plants harbor endophytic fungi belonging to several groups, such as *Acremonium* spp., *Colletotrichum gloeosporioides*, *Fusarium*, *Gliocladium*, *Lasiodiplodia theobromae*, *Pestalotiopsis* spp., *Trichoderma* spp. and *Verticillium* spp. [50]. *Fusarium* sp. and *Colletotrichum* sp. were also isolated as endophytes from cacao leaves in Panama [51]. The most frequent endophytically isolated fungi include *Colletotrichum, Cladosporium, Fusarium* and *Xylaria* species [52,53,54,55]. 

The diversity of plant endophytes from *Paeonia* spp. was recently analyzed and different genera of fungi were identified. The most abundant among them were *Fusarium, Phoma, Alternaria* and *Pestalotiopsis* [56]. Other examples can also be found, such as *Coniothyrium* species isolated from the cortex of *Picea abies* branches [57], *Asteromella* fungus isolated from the inside of *Quercus emoryi* leaves [58], *Phoma* isolated from wheat leaves [59] and *Aspergillus, Curvularia lunata, Fusarium, Penicillium* and *Trichoderma* isolated from sunflower seeds [60]. In addition, there were a total of 60 isolates of endophytic fungi belonging to 16 different genera in the medicinal *Sceletium tortuosum* plant in South Africa, the most ubiquitous being the *Fusarium*, *Aspergillus*, *Penicillium* and *Phomopsis* genera [61]. Yerba mate plants (*Ilex paraguariensis*) are also colonized by endophytic fungi, with the main ones being *Aspergillus*, *Penicilium*, *Acremonium*, *Fusarium* and *Colletotrichum* [62].

The population dynamics of endophytic fungi may be related to some host properties such as chemical composition [63], physiological conditions [64], geographic distribution, plant age and ecological conditions, including altitude and precipitation [65]. Ecological or environmental conditions such as temperature, humidity, lighting, geographic location and vegetation significantly affect the distribution pattern and population structure of endophytic fungi [56]. For example, one or two species are predominantly endophytic in a given host, while others are uncommon [51,66]. The population of endophytes of a given plant can also vary according to their health state, suggesting that the microorganisms have a probable protective action [67,68].

Although endophytes are closely related to the plants, they need to overcome the defense barriers interposed. For example, secondary plant metabolites are one of these obstacles for colonization by endophytic fungi and, therefore, these organisms must secrete corresponding detoxifying enzymes. Thus, they are able to decompose the secondary metabolites so that they can enter and colonize the host plant tissues. In addition, these detoxifying compounds secreted by endophytic fungi can in turn induce production of a variety of new bioactive secondary metabolites, which can further serve as important medicinal resources [69].

With all the existing evidence, endophytes have begun to be recognized for their ability to protect their hosts from pathogens and be used as biocontrol agents. Thus, isolating and characterizing endophytic microorganisms from plants which have not yet been studied can enable the discovery of new species with the potential to produce substances of interest such as compounds with antimicrobial activity, which are extremely important for industry [40]. In addition, the ability of in vitro production of substances which inhibit the growth of other microorganism species has stimulated research regarding the bioprospecting of endophytic fungi for biological control [70].

## 3. Biological Control

As stated, plant pathogens always threaten world food security. In many cases, the available tools have not been enough to properly manage them and reduce losses. For example, *Phytophthora infestans* was the first plant pathogen successfully reported by De Barry in 1845 [28], but still constrains the production of important crops such as tomato and potato [71,72]. We can also highlight plant parasitic nematodes. A single species, the root-knot nematode (*Meloidogyne incognita*), presents one of the broadest host ranges among all plant pathogens, being able to parasitize more than 3000 plants [73]. It damages and imposes limitations to food and plant-resource production in both tropical and temperate areas of the world. Despite the phylogenetic differences (an oomycete and an invertebrate animal, respectively), they share some similarities from a management perspective. Together, the broad range of hosts and the rise of virulent strains/populations impose difficulties in the use of resistant cultivars and non-host crops. The use of chemical pesticides is not always efficient (insensitive strains) or viable (cost or application method). In addition, the pesticide industry has been struggling to produce novel pesticide molecules. Lastly, society not only demands security in food production, but higher quality and lower impacts on the environment [74]. Altogether, these factors have driven the search for novel, effective and eco-friendly ways to manage pests, which has enabled biopesticides to become an important asset to reduce losses from plant pathogens. In view of the above, then, what is biological control? Traditionally, biological control is defined as a decrease in a pathogen population (inoculum) or in the disease determinants by an organism which is not human or plant [75]. It is also referenced as an attempt to transport a common phenomenon from nature to the agricultural systems, taking advantage of natural and established relationships [76]. However, most (if not all) biological control agents have demonstrated the ability to closely interact and/or colonize plants in some way. They developed a complex inter-kingdom communication in which signaling occurs through a biochemical language with plants [77,78,79]. For example, plants have the ability to harbor a microbial community in the rhizosphere, being able to recruit some in unfavorable situations [80]. This current view of biological control especially mediated by endophytes opens a novel way to face microorganism–plant relationships and unveil new biotechnological tools to manage plant pathogens. We will address this subject in the present section.

Biological control is a wide and generic category which embraces relatives and distant phylogenetic organisms, as well as different suppression mechanisms of plant pathogens. There are several invertebrates (e.g., acari, predatory nematodes, parasitoids, tardigrades), fungi (e.g., avirulent strains of *Fusarium*, *Trichoderma*), bacteria (*Bacillus* spp., *Pseudomonas* spp.) and viruses among biological control agents. However, not all of them are suited to be used as biopesticides as they have to fulfill several requirements, among which we would like to highlight the following: (1) they are not harmful to plants, humans or animals; (2) Are efficient in controlling the target; (3) they survive in different conditions and in the absence of the target; (4) they are economically viable to produce on a large scale; (4) they have a long shelf-life and are infective after being stored; (5) they are compatible with different agricultural assets (pesticides, fertilizers, etc.) [81,82,83,84,85].

Taking these requirements into consideration, the most prominent agents to control plant pathogens are fungi and bacteria. As stated, most of them have the ability to colonize plants.

Regarding the action mechanisms, biological agents can suppress pathogens through predation, parasitism (sometimes referred to as hyperparasitism, the parasite of a parasite), secretion of repellent and/or toxic compounds, including volatiles (antibiosis) and competition for a specific niche (nutrient, infection site, plant tissues, etc.). 

The same agent often uses several mechanisms at the same time or applies different mechanisms for different pathogens. For example, *T. harzianum* usually antagonizes *Sclerotinia sclerotiorum* through direct parasitism, in which *Trichoderma* coils and degrades the target’s hyphae [86]. It can also inhibit a white mold agent through antibiosis and competition for space and/or resources [87]. Another interesting example is *Purpureocillium lilacinum* (syn. *Paecilomyces lilacinus*), a fungus known for its effect against plant parasitic nematodes. *P. lilacinum* performs antibiosis against *S. sclerotiorum*, and thus antagonizes it by producing and secreting an array of extracellular enzymes which inhibit the development of the white mold agent in vitro [88]. It also parasitizes the eggs and egg-laying females of root-knot nematodes (*Meloidogyne* spp.) by killing and digesting them with extracellular enzymes [89,90]. These examples emphasize that the most important suppression component can change with the pathosystem: to the white mold agent hyperparasitism and antibiosis for *T. harzianum* and *P. lilacinum*, respectively. In contrast, regarding the root-knot nematode, antibiosis and hyperparasitism by *T. harzianum* and *P. lilacinum*, respectively. 

Some biological agents colonize the plant, which often present biostimulating effects. Baron et al. [91] showed that *P. lilacinum* and *Metarhizium marquandii* promote growth in maize, bean and soybean plants when used as bioinoculants. They observed indoleacetic acid (IAA) production and phosphorus solubilization, showing the biostimulating effect of these endophytes in addition to their effects against plant pathogens. The biological control mediated by endophytes and their beneficial effects on plants will be further addressed in the specific section of the present review.

Although biopesticides are currently commercialized worldwide, the development and the subsequent steps (i.e., packing and shelf-life) of biological control agents are not easily carried out. A discrepancy in results obtained in controlled field conditions are often reported among the main limitations. Interference from the environment is usually overcome in laboratory conditions [92] and sometimes could lead to misleading conclusions [93]. In addition, another issue is the ineffectiveness of the biopesticide in different environmental conditions and in population variations of the plant pathogens. The effectiveness of biopesticides may vary among cultivars of a particular host. 

Biological control does not follow the same pattern as chemical pesticides. The use of biopesticides is complex and is influenced by the environment and agricultural conditions. Biological agents do not aim to eradicate pathogens. Instead, their use is intended to reduce them to non-harmful levels, below the dangerous threshold [15,94]. Even so, the use of biopesticides presents several (already presented) advantages which we will further develop focusing on the potential of endophytes in agriculture.

## 4. Induced Resistance

Plant resistance can be defined as the ability of the host to delay and/or prevent colonization and development of the pathogen. There are several defense mechanisms involved in resistance, which can be biochemical or structural, and still classified on a temporal scale as preformed or postformed [85].

According to Kesel et al. [95], the plants have an immune system composed of constitutive and inducible defenses which can be increased through biotic and/or abiotic stimuli, providing higher defense capacity against pathogens and pests, characterizing the induced resistance. Thus, this phenomenon in plants can be seen as a possible measure for controlling plant diseases, in addition to being used as a tool for studying the resistance mechanisms and susceptibility of plants against phytopathogens [17,96,97,98].

The induced resistance has several advantages; for example, it can be effective against viruses, bacteria, fungi, phytonematodes and abiotic stresses. In addition, it exhibits stability due to the fact that different resistance mechanisms work together, highlighting the non-specificity, systemicity, persistence, and grafting transmission, among others [85].

The most desired result in induced resistance is the state of “priming”, in which the elicited plants go into a “state of alert”, and the resistance mechanisms are more intensely expressed with the arrival of the stressor, and to a lesser extent time lapse. However, this state does not result in energy expenditure due to the latent state of the mechanisms that govern resistance [85,99,100].

The term induced resistance can be used to designate a local protection only in the tissues where the treatment with the inducing agent was carried out, but it can also indicate a systemic resistance which manifests itself at a distance from the inducer application site [101,102]. 

Activation of plant defense can occur from elicitation by compounds present in plant extracts [103,104,105], yeast preparations [106,107], growth-promoting rhizobacteria [108], growth-promoting fungi [109], avirulent pathogens [110], endophytic fungi [47,111,112], among others.

Therefore, induced resistance consists of activating resistance through the use of external agents without any change in the plant’s genome [97], and non-specifically occurring through the activation of genes involved in several defense responses such as oxidative explosions [113], hypersensitivity responses [114], accumulation of PR-proteins [115], enzymes involved in the phenylpropanoid pathway [116,117], enzymes involved in lipid peroxidation [118], phytoalexin synthesis [119], and accumulation of phenolic compounds [120], among others.

According to the signaling pathway which promotes the expression of defenses, induced resistance can be divided into resistance induced by non-pathogenic microorganisms and biotrophic pathogens which have salicylic acid (SA) as the main signaling agent, mainly expressing PR-proteins, and designated as systemic acquired resistance (SAR). The resistance induced by rhizospheric growth-promoting microorganisms or necrotrophic pathogens, known as induced systemic resistance (ISR), has jasmonic acid (JA) and ethylene (ET) as the main signaling agents, independent of SA [121,122,123,124].

This is a generalization, since there are already reports where the pathogen *Phakopsora pachyrhizi*, the causal agent of Asian soybean rust, supposedly modulates the expression of target genes when penetrating the host tissue, activating the JA pathway and inhibiting the defense mediated by SA [125]. It is believed that there is a positive crosstalk between SA, JA, and ET, in addition to gene expression effectors. In a study using *Arabidopsis* isolated rhizobacteria, it was shown that the SA and JA pathway has additive effects on the induced resistance against the *P. syringae* pv. *tomato* pathogen. It is believed that the responses mediated by SA and JA are capable of working together to a certain degree, with the prevalence of one over the other after a certain time [126,127].

## 5. Endophytes as Biocontrol Agents

The biological control of plant pests has been boosted in recent years. As discussed, the agents have demonstrated the ability to colonize plants or at least to establish a close relationship with them. Thus, most biological control endophyte (BCE) agents have frequently been found among different crops and are able to suppress important pathogens (Table 1).

Several *Trichoderma* species are among the most studied BCE. *Trichoderma* species are able to colonize a wide range of crops such as soybean, wheat, corn and cotton. This fungus has shown different mechanisms involved in disease control and is widely known for its capacity to induce resistance in plants [128], although it shows a remarkable ability to parasitize very different plant pathogens. For example, *Trichoderma* spp. have been found to parasitize *S. sclerotiorum* and *Rhizoctonia solani* hyphae [129]. In addition, several strains have been reported penetrating and parasitizing eggs and second-stage *M. javanica* juveniles and *Heterodera avenae* cysts, a very resilient structure [130,131]. Thus, the *Trichoderma* species present a wide number of hosts which include both plants and the different plant pathogens as symbiotic and parasitic, respectively.

In addition to the direct parasitism, several BCE produce and release many effector compounds (e.g., antibiotics, toxins and fungitoxic metabolites) against plant pathogens. BCEs inhibit pathogens through the production of cellulase, glucanase, chitinase and lactones (volatile compounds) [132]. This kind of mechanism is also observed in other endophytes. For example, *P. lilacinum* is a classic biocontrol agent of plant parasitic nematodes and insects, but have demonstrated the ability to suppress different plant pathogens through deploying effectors. Wang et al. [133] showed the involvement of the leucinostatins (antibiotics) on the suppression of *P. infestans* and *P. capsici*. The culture filtrates of *P. lilacinum*, which contains effectors, suppressed *S. sclerotiorum* and induced defense responses in the common bean [88]. Furthermore, the culture filtrate and cell wall extract of *Piriformospora indica* reduced the infection of *Heterodera schachtii* in *Arabidopsis* based upon nematode per cm of root, syncytia length and eggs per cyst in [134]. This fungus interestingly does not parasitize the nematode. These examples highlight the potential of the cell-free filtrates of BCE to control plant pathogens in agriculture nowadays.

Another group of prominent endophytes are the non-pathogenic strains of plant pathogens. *F. oxysporum* sensu lato can interact with plants as a pathogen, causing root rot or wilt. However, some avirulent strains of *F. oxysporum* sensu lato can colonize plants as endophytes and protect the plants against soil-borne diseases (i.e., *Pythium ultimum* and *Verticillium dahliae*) [135]. *F. oxysporum* sensu lato can also compete for nutrient and/or root niches, which suppresses fungal plant pathogens [135,136]. However, other mechanisms may be involved such as *Fusarium* endophytes which controlled *F. oxysporum* f. sp. *lycopersici* in tomato plants through induced resistance mediated by SA, JA and ET [137]. Induced resistance mediated by endophytes will be further discussed in the specific section below.

Some studies have interestingly shown the potential of non-toxigenic strains of *Aspergillus flavus* on the control of mycotoxigenic *Aspergillus* in cereals [138]. The strategy to avoid aflatoxin contamination at a pre-harvest stage includes introducing the non-pathogenic *A. flavus* strains to compete and suppress the toxigenic *Aspergillus* [138]. Additionally, other biocontrol agents have similarly been used to control toxigenic strains of *Fusarium* in maize [138,139].

As a result, the following question arises: how can we isolate and select potential fungal endophytes to control plant pathogens? The potential answer may be the plant’s biome. The microbes associated to plants have been demonstrated to be effective to control most pathogens related to this host crop. For example, Halecker et al. [140] aimed to develop a biocontrol agent by using an endophyte fungus to control ash dieback caused by *Hymenoscyphus fraxineus*. A total of 340 endophytic fungi were isolated from the *Fraxinus excelsior*, the tree host. The fungi were further investigated and co-cultivated to find a suitable biocontrol agent. Rubini et al. [50] investigated the fungal community of cacao plants (*Theobroma cacao*) and addressed the biological control of *Moniliophthora perniciosa*, the causal agent of witches’ broom disease. A diverse number of fungal genera were found associated to cacao plants, but only one reduced the incidence of the disease: treatment with *Gliocladium catenulatum* reduced the incidence of witches’ broom disease in 70% of the infected plants. This highlights the potential of the phytobiome to be used in the control of plant pathogens. Additionally, despite not being the focus of the present review, the potential of bacterial endophytes is noteworthy. Similar to what has been discussed, Khaskheli et al. [141] addressed the importance of root-associated bacterial endophytes from rice plants to control its major diseases. They followed a similar approach and we recommend their work for additional material.

Thus, given the relevance of endophytic fungi for controlling plant diseases, Table 1 presents an overview of the scientific work carried out with endophytic fungi in the area of biological control regarding phytopathogens.

**Table 1:** Phytopathogens affected by endophytic fungi based on mechanisms related to biological control *.

Endophytic Fungi	Plants	Fungi Targets	References
*Cladosporium tenuissimum*	-	*Uromyces appendiculatus*	[142]
*Trichoderma viride*, *T. harzianum*, *T. stromaticum*, *T. virens*	-	*Rhizopus stolonifer*	[143]
*Trichoderma viride*	-	*Penicillium digitatum*	[144]
*Trichoderma viride*	-	*Phytophthora nicotianae*	[132]
*Trichoderma viride*	-	*Rhizoctonia solani*	[145]
*Trichoderma viride, T. koningii*	-	*Verticillium dahliae*	[146]
*Fusarium oxysporum* sensu lato	*Solanum lycopersicum*	*Phytophthora infestans* and *P. capsici*	[23]
*Xylaria* sp.	*Ginkgo biloba*	*Penicillium expansum* and *Aspergillus niger*	[36]
*Heteroconium chaetospira*	*Brassica oleracea*	*Verticillium dahliae*	[147]
*Diaporthe helianthi*	*Leuhea divaricata*	*Moniliophthora perniciosa*	[148]
*Aspergillus*, *Penicillium* and *Trichoderma* sp.	*Eucalyptus benthamii*	*Botrytis cinerea*	[149]
*Trichophyton* sp., *Chrysosporium* sp., *Candida pseudotropicalis*, and *Candida tropicalis*	*Symphytum officinale*	*Sclerotinia sclerotiorum*	[150]
*Colletotrichum gloeosporioides* and *Clonostachys rosea*	*Theobroma cacao*	*Phythophthora* sp. and *Moniliophthora roreri*	[151]
*Gliocladium catenulatum*	*Theobroma cacao*	*Crinipellis perniciosa*	[50]
*Diaporthe terebinthifolii*	*Schinus terebinthifolius*	*Phyllosticta citricarpa*	[152]
*Fusarium solani* sensu lato	*Vitis labrusca*	*Botrytis* sp.	[153]
*Aspergillus insulicola* and *A. melleus*	*Sesuvium portulacastrum*	*Pythium aphanidermatum*	[154]
*Phyllosticta fallopiae*	*Cornus officinalis*	*Alternaria alternata*, *A. arborescens*, *Botryosphaeria dothidea* and *Colletotrichum gloeosporioides*	[155]
*Alternaria tenuissima*	*C. officinalis*	*Alternaria alternata*	[155]
*Alternaria alternata*	*C. officinalis*	*Alternaria arborescens*	[155]
*Botryosphaeria dothidea*	*C. officinalis*	*Alternaria alternata*, *A. arborescens*, *Botryosphaeria dothidea* and *Colletotrichum gloeosporioides*	[155]
*Colletotrichum gloeosporioides*	*C. officinalis*	*Alternaria alternata*	[155]
*Botryosphaeria berengeriana*	*C. officinalis*	*Botryosphaeria dothidea*	[155]
*Alternaria* sp., *Botryosphaeria ribis*, *Phoma medicaginis*, *Bionectria ochroleuca*, *Aureobasidium pullulans* and *Chaetomium* *spirochaete*	*Vitis vinifera*	*Botrytis cinerea*	[156]
*Ramularia pratensis, Phoma aliena* and *Fusarium acuminatum*	*Vitis riparia*	*Botrytis cinerea*	[157]
		**Bacteria targets**	
*Xylariales* sp.	*Distylium chinense*	*Clavibacter michiganensis*, *Xanthomonas citri* pv. *phaseoli* var. *fuscans* and *Pseudomonas syringae* pv. *lachrymans*	[158]
		**Viruses targets**	
*Paecilomyces variotii*	*Nicotiana benthamiana* and *N. tabacum*	*Potato Virus X* (PVX) and *Tobacco mosaic virus* (TMV)	[159]
		**Nematodes targets**	
*Acremonium implicatum*	*Solanum lycopersicum*	*Meloidogyne incognita*	[160]
*Fusarium oxysporum* sensu lato	*Musa* spp.	*Pratylenchus goodeyi*	[161]
*Chaetomium globosum*	-	*Meloidogyne incognita*	
*Daldinia* cf. *concentrica*	*Olea europaea*	*Meloidogyne javanica*	[162]
*Alternaria* sp.	-	*Bursaphelenchus xylophilus*	[143]

* The possible mechanisms of action are commented on in the text. “-” means that the host plant was not identified in the cited reference. The Bold is applied to divide different kinds of plant pathogens.

## 6. Endophytes in Induced Resistance

The presence of endophytic fungi in plants can induce them to produce compounds which act on phytopathogens or alter their plant morphology so that they may be better able to defend themselves in unfavorable situations. The action mechanisms of endophytes in inducing resistance may include increased synthesis of phytoalexins and PR-proteins, cell wall thickening through depositing lignin and glucans, increased cuticle thickness, among others, which may hinder penetration and development of the pathogen in the host plant [47].

The endophytic microorganisms have the ability to produce a large number of secondary metabolites, with this number being higher than any other microorganism [69]. It was recently revealed that the endophyte-plant interaction can go beyond the balance between virulence and defense, being much more complex and precisely controlled [163]. Among the control mechanisms provided by endophytes such as competition for space and nutrients, mycoparasitism, antibiosis and induced resistance, there is a high probability that induced resistance is one of the most important mechanisms used by endophytes in disease control [23]. Some of the compounds recognized by the plant are common among all fungi, such as certain cell wall components and enzymes such as xylanases, cellulases and chitinases [163]. Other compounds are more specific for certain species, including secreted proteins, specialized metabolites and lipids, hormonal molecules and volatile compounds [164].

Some studies report the production of bioactive molecules by endophytic microorganisms identical to those produced by the host plant [165]. These studies corroborate the theory that they adapted to the plant microenvironment during the co-evolution of the host plant with the microorganism and were able to assimilate part of their hosts’ DNA to their genome, acquiring the ability to synthesize bioactive compounds [166]. Other theories assume that the reverse is also true, so that part of the microbial DNA was assimilated to the plant’s genome during a co-evolution process, and what was exclusive to the endophyte is also passed to its host [167]. Thus, endophytic fungi can regulate biochemical routes, leading to the production of substances which are common to their hosts or vice versa, and which can have applications outside the plant in which they live [168]. Examples of endophytic microorganisms that produced the same metabolites as the host plant can be illustrated by *Fusarium* sp. and *Myrothecium* sp. fungi [169], as well as macrocyclic trichothecene producers, which were isolated from *Baccharis megapotamica* and *B. coridifolia* plants [170,171].

*Gilmaniella* sp. is an endophytic fungus isolated from *Atractylodes lancea* plants, and has been reported to produce metabolites with an elicitor effect on its hosts which can substantially improve the total volatile oil content, while in turn the fungus could effectively improve the quality of herbal medicines [172]. Endophytes isolated from *Cicer arietinum* plants have been identified and characterized due to their ability to induce resistance in plants by producing higher levels of defense compounds, antioxidant and phenolic enzymes, in addition to solubilizing P and Zn, and reducing infection by *B. cinerea* in plant tissues [173]. The moderate and constant activation of these enzymes can be a key mechanism for plant resistance [173].

The endophytic fungus *P. indica* has a wide range of hosts and exhibits interesting biological activities for agriculture such as promoting growth, inducing resistance against phytopathogens, water and abiotic stresses, among others [174]. For example, *P. indica* induces resistance against *Fusarium* in *Hordeum vulgare* [175], *T. aestivum* [176], *Z. mays* [177] and *S. lycopersicum* [178] plants. Endophytic fungi may present systemic distribution in the plant or be restricted to certain tissues such as the roots and stem, among others. In this sense, the inoculation of *Blumeria graminis* in *H. vulgare* plants and the pre-inoculation of *P. indica* in the root system reduces 58% of the symptoms of the disease, clearly demonstrating the promotion of induced resistance [175].

The SA-dependent defenses are generally effective against biotrophic pathogens, while JA/ET-dependent defenses are effective against necrotrophic pathogens [179,180,181,182]. Thus, it is assumed that if an endophyte tends to increase protection against necrotrophic fungi and makes the plant resistant, on the other hand it may become more susceptible to biotrophic fungi [183].

**Figure 2:**
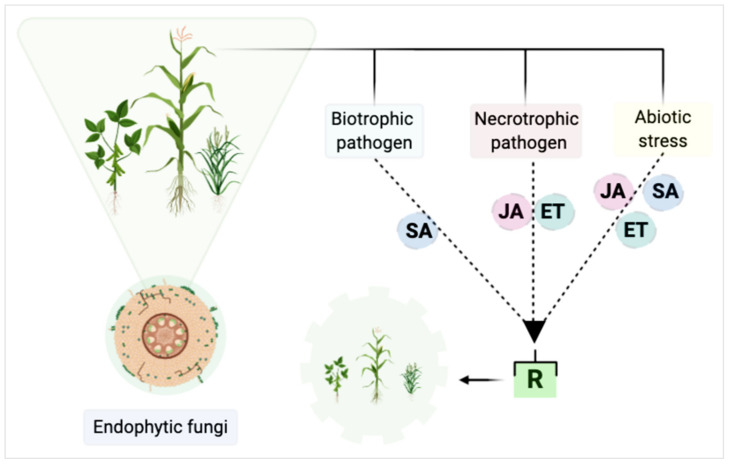
Main plant pathways triggered in defense processes using fungi and abiotic stresses as models. SA—salicylic acid; JA—jasmonic acid; ET—ethylene; R—resistance. Adapted from Bastias et al. [184], with additional information from Thlaer et al., Kunkel & Brooks, and Junt et al. [179,180,183]. Created with BioRender.com.

The suppression of plant diseases in most cases occurs by manipulating the JA and ET pathway by beneficial microorganisms leading to induced systemic resistance (ISR) [174]. Despite this, other hormones may be involved in the phenomenon of induced resistance, however, they will not be discussed here. Based on this information, it is possible to differentiate the defense mechanisms of the plant when it induces resistance to fungi or abiotic stresses (Figure 2). If a plant shows infection with biotrophic fungi, signaling will normally occur from the salicylic acid pathway (Figure 2). However, if the infection occurs from necrotrophic fungi, signaling occurs via the JA and ET pathways. Induced systemic resistance can promote local or systemic resistance of the plant against biotrophic fungi, for example, and susceptibility to necrotrophic fungi, making the plant resistant or susceptible depending on the triggered pathway (Figure 2).

The triggered metabolic pathways, as seen in Figure 2, are dependent upon which microorganism will affect the plant, and although the benefits of endophytic fungi in plant development are elucidated, the mechanisms involved in the plant vs. endophytic vs. pathogen/abiotic interaction are not well understood.

It should be noted that the crosstalk between SA, JA and ET signaling enables the plant to fine-tune the defense response [121]. For example, systemic resistance dependent on JA/ET has been found for some endophytes such as *Piriformospora indica* [185]. However, *P. indica* induced resistance independent of the JA/ET pathway in other pathosystems. These findings indicate that the hormonal roles and their interactions are complex, and the application of a microorganism to the plant probably alters the entire hormonal profile, depending on the host and the inducing agent.

When evaluating the compounds produced by chickpea plants inoculated with endophytes, a high production of indole acetic acid (IAA) was found [111]. It is already known that IAA levels contribute to higher growth of sprouts and roots [186], for example, mandarin plants inoculated by endophytes such as *Nocardia*, *Nocardiopsis*, *Spirillospora*, *Microbispora* and *Micromonospora* have higher length, number of shoots and root mass.

An avirulent isolate of *F. solani* sensu lato obtained from the tissues of *C. acuminata* bark, has been reported as a producer of the metabolite camptothecin, which guarantees its protection against this compound through specific changes in the catalytic domains of its topoisomerase I [187]. Likewise, topoisomerase I encoded by other endophytic fungi, isolated from the same tissue, but which does not produce camptothecin, contains the same changes to make it resistant to camptothecin action. This suggests that evolutionary pre-adaptation is similar in endophytes which infect the same plant, regardless of its biosynthetic capacity [188], ensuring that endophytic microorganisms have positive interactions and that their metabolites are not toxic to their hosts.

Given the above, an overview of the scientific work carried out with endophytic fungi to induce resistance can be seen in Table 2.

## 7. Endophytes in Inducing Tolerance to Abiotic Stresses

Endophytes have been used as sources of biotic elicitors because of their ability to simulate responses to diseases in plant cells. Endophytes have stood out for their ability to synthesize and accumulate secondary metabolites in the tissues of their hosts which can influence the functioning of antioxidant enzymes, in turn activating the cascade of defense signals and promoting the positive regulation of gene expression of important enzymes during the production of secondary metabolites [221]. In this sense, several studies have shown that the association of endophytes increases tolerance to abiotic stresses [221,222,223,224,225,226].

There is currently a need for new agricultural practices to maximize the efficiency of crops at elevated temperatures due to the increasing effects of global climate changes [226]. The ability of endophytes to confer heat tolerance has been observed in plants such as *Adiantum capillus-veneris* [227], *Helianthus annuus* and *Glycine max* [228], *Cucumis sativus* [226], among others.

Treatment with the thermophilic *Thermomyces* sp. endophytic fungus which supports high temperatures (CpE) eliminated the adverse effects of thermal stress on cucumber plants, maintaining the maximum quantum efficiency of photosystem II, the photosynthesis rate and water use efficiency. In addition, CpE treatments induced significant accumulation of total sugars, flavonoids, saponins, soluble proteins and the activities of antioxidant enzyme compared to untreated cucumber plants under heat stress conditions [226]. On the other hand, cucumber plants treated with *Thermomyces* sp. exhibited an improvement in root length over untreated cucumber plants. This phenological response is an essential adaptive trait in desert ecosystems, enabling the plant to better penetrate and extract soil moisture and nutrients under limited water conditions [226].

Plants under thermal stresses quickly increase stomatal conductance, thereby promoting a high transpiration rate. Even under these conditions, these plants have a slow stomatal opening and a low transpiration rate when they are treated with endophytes [226]. The endophytic *Thermomyces* sp. maintained water content in the leaf, increasing the water use efficiency under stress conditions. In addition, thermophilic fungi prevent excessive water losses from the plant through stomatal closure as a physiological-adaptive strategy to save water before further damage occurs due to increased temperature stresses [226]. These fungi promote an accumulation of primary and secondary metabolites [226]. The higher accumulation of sugars and flavonoids in plant tissues in many plant-microbe interactions act as reactive oxygen species (ROS) scavengers and signaling molecules, thereby enabling plant growth and tolerance to abiotic and biotic stresses [229].

The role of endophytes in providing tolerance to water stress by regulating stress-inducible genes has been reported in *Cucumis sativus* [230], *Zea mays* [231,232], *Oryza sativa* [233], *S. lycopersicum* [234], *Triticum aestivum* [235], *Citrus reticulata* [225] and *Saccharum officinarum* [236]. The relief of water stress due to the action of endophytes may be the result of an increase in antioxidant enzymes, bioactive compounds, chlorophyll content, carotenoid content and chlorophyll fluorescence. In addition to changing all these parameters in *C. reticulata* plants, *Penicillium citrinum, Aureobasidium pullulans* and *Dothideomycetes* sp. endophytes also promoted plant growth [225].

The mechanisms mediated by endophytes are reported to facilitate plant adaptation to drought tolerance by generating phytohormones, ROS, exopolysaccharides, 1-aminocyclopropane-1-carboxylate deaminase, and volatile compounds; change in root morphology; biosynthesis of anti-stress metabolites and positive regulation of stress-responsive genes in host plants [237]. In addition, the accumulation of solutes in plants with endophytes is reported in grasses when subjected to water stress [238].

One of the hypotheses for tolerance to water stresses mediated by endophytes in host plants is the use of CO_2_ released by endophytes to continue photosynthesis. This relieves the lack of CO_2_ in stressed plants due to stoma closure. It was reported that 2.7% of CO_2_ released in the roots by endophytes in *Populus deltoides* was assimilated in the host’s photosynthesis [239].

The role of endophytes in providing tolerance to heavy metal stresses has been observed in plant cultures such as *Triticum aestivum* [224,240], *Lycopersicon esculentum* [241], and *Glycine max* [242], among others. For example, the endophytic *P. roqueforti* fungus induced resistance in *T. aestivum* plants grown in soil contaminated with heavy metals, restricting heavy metal transfer from the soil to the plants, and secreting indole acetic acid. In addition, these wheat plants inoculated with the endophytic fungus and watered with residual water showed higher growth, nutrient absorption and low heavy metal concentrations in the shoot and roots. In contrast, wheat plants not inoculated under heavy metal stress showed stunted growth with chlorosis symptoms. The inoculation of *P. roqueforti* can establish a symbiotic relationship with host plants, which is useful for stabilizing heavy metals, meaning that it helps host plants to flourish in soil that is highly contaminated with heavy metals [224]. Thus, the endophytic fungi increase the host plant’s capacity to accumulate heavy metals by direct or indirect mechanisms in addition to cell detoxification by enzymatic activity. Endophytes can directly help the host plant through increased mobilization of heavy metals, thus alleviating the toxicity level of metals in plants [243], or indirectly by improving plant growth and stress tolerance.

**Figure 3:**
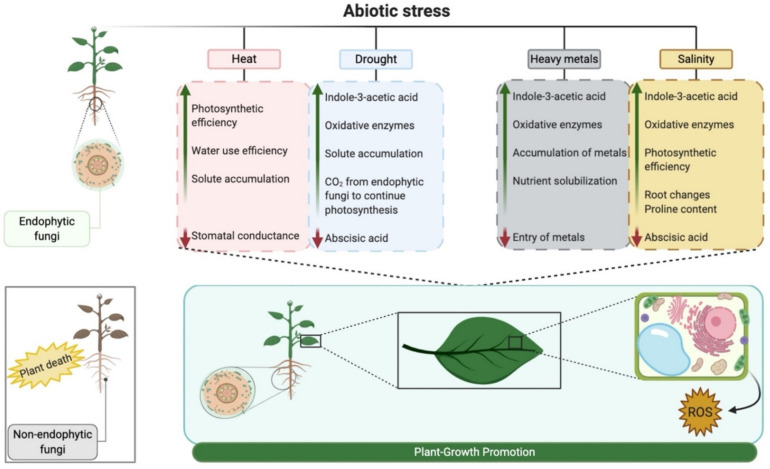
Potential reactions to abiotic stresses evidenced by plants when interacting with endophytic fungi. The green arrow represents the increase and the red arrow represents the reduction of the listed characteristics. Created with BioRender.com.

Endophytes can benefit the host plant by increasing its ability to absorb essential nutrients from contaminated soil [244]. Furthermore, these fungi can degrade pollutants present in contaminated soil [245] and convert them to a non-toxic form. The exogenous supply of phytohormones by endophytes can bring positive physiological changes in the host plant to withstand stress conditions. In addition to phytohormones, the biofertilization capacity of endophytic fungi can increase the availability of nutrients to the host plant in soil contaminated with heavy metals through solubilization [246]. The possible mechanisms modified by the interaction with endophytic fungi under abiotic stresses can be seen in Figure 3.

The role of these microorganisms in providing tolerance to salt stress has been observed in plant cultures such as *Z. mays* [222], *S. lycopersicum* [247], *O. sativa* [248], *T. aestivum* [249], *Cucumis sativus* [230], and *G. max* [223,250], among others. For example, the endophytic fungus *P. indica* increased the growth and yield of *S. lycopersicum* under salt stress conditions, inducing a series of morphological and biochemical events which together contributed to relieve the impact of salt stress. This endophyte promoted an increase in the chlorophyll and indole acetic acid content, enzymes such as catalase and superoxide dismutase, increased the root branching, the fresh and dry mass of plants and fruit production by 65% under salt stress. In addition, tomato plants colonized with endophytes reduced abscisic acid (ABA) and proline levels when compared to non-colonized plants [247]. The ROS-sequestering enzymes appear to substantially contribute to improving salt stress tolerance [251].

Many plants produce high proline levels under salt stress; however, these proline levels can be reduced when plants are inoculated with endophytic fungi [247]. ABA controls proline biosynthesis to reduce cytoplasmic osmotic stress caused by increased salts in the root zone [252] and, therefore, for example, ABA levels are reduced by approximately 30% under saline stress conditions, and the proline content is consequently reduced [253].

Abiotic stresses, including oxidative stress, drought, flooding, salinity and heat stress are interrelated, resulting in the synthesis of ROS which cause cell damage, and consequently cell death under prolonged exposure [230]. An increase in the amount of ROS in plant cells causes oxidative degradation of RNA and DNA, lipid peroxidation and oxidative stress [254]. The ROS signal directly modifies the redox balance of regulatory proteins, transcription and translation, thereby stimulating responses in the plant which help to reduce the negative effects of stress and moderate the metabolic ROS concentration [255].

The hypothesis is that endophytes also initially secrete a small amount of ROS, for example hydrogen peroxide, which triggers the antioxidant enzymes of the infected host [256]. The constant release of ROS in small amounts prevents cell hypersensitivity to ROS, improves the absorption of nutrients (calcium, potassium, magnesium and phosphorus) by plants and increases other endosymbiotic interactions of the host [257]. One of the main responses by plant tissues to the presence of ROS produced by endophytes is to accumulate proline, methionine, flavonoids and other phenolic compounds to increase their resistance [258].

The probable mechanisms by which hypersensitivity responses and acquired systemic resistance of the hosts can occur involve the crosstalk between endophytes and host plants, as well as the generation of ROS and antioxidants [259]. While some fungal endophytes produce ROS to acquire nutrients from host cells and maintain their mutualistic interactions with plants, other fungal endophytes lower ROS concentrations to mitigate the effect of abiotic stresses on their hosts [260].

Based on the above, a general view of the scientific work carried out with endophytic fungi exhibiting effects on abiotic stresses can be seen in Table 3, together with the possible altered mechanisms outlined in Figure 3.

## 8. Perspectives

It is known that each of the approximately 300,000 species of plants existing on Earth includes a universe of endophytic microorganisms, especially woody plants, which may contain numerous species with potential for studies [267]. Elucidating and identifying the most active metabolite structures are essential to develop new products [268]. It is worth considering that individual substances of a crude extract often do not present relevant microbial activity, since the compounds present in this extract act synergistically with other substances produced by the microorganism [268]. Thus, elucidating the action mechanisms of endophytic fungi and their interaction in plant protection, either by the action of direct biological control, or by induced resistance and tolerance to abiotic stresses, make endophytic fungi a highly promising tool for inserting into integrated management, and widely important for the agribusiness.

During the course of evolution, the endophytes were not only able to colonize plants, but developed a complex signalization with their hosts, promoting benefits which could be explored in agricultural systems. The signalization is mediated by effector molecules, usually proteins that are delivered to the host plant and trigger beneficial effects, e.g., growth promotion and induced resistance. Several studies have been dedicating their efforts to understand this intricate network by using different approaches, notably the Omics approach (genomics, transcriptomics, proteomics, metabolomics, etc.) [269,270]. The Omics approach offers the possibility to identify and characterize proteins and genes, which could be useful to select promising strains as biopesticides, plant growth promoters, etc. In addition, metagenomic analyses have been used to investigate the microbial diversity associated with plants under several environmental conditions [271]. Furthermore, metagenomic analyses enable investigating the microbiome in plant health in crops and natural system [271]. Although the focus of the current review is not the Omics approach, it is important for understanding the endophyte–plant interactions and their possible use in agriculture. Finally, society’s pressure for food production in more sustainable ways with biotechnological approaches is encouraging exploitation of endophytic microorganisms.

## 9. Conclusions

Endophytic fungi can trigger innumerable mechanisms in the plant, providing protection against biotic and abiotic disorders. These fungi satisfactorily perform biological control against plant diseases with the potential to be used as a tool for bioprospecting new molecules and genetic modification of plants due to their potential for genetic modulation and interaction with the host.

Tolerance to abiotic stresses can be obtained by an association of endophytes with the target cultures, presenting promising results and making it possible to grow plants in certain places where plants without association with the endophytic agent could have difficulties to developing.

The secondary metabolites produced by endophytes exhibit important biological activity and can become valuable products. Thus, isolating and characterizing endophytic microorganisms from plants which have not yet been studied can enable discovering new species with the potential to produce substances of interest which can be used in the biological control of diseases, as elicitors in induced resistance and for inducing tolerance to abiotic stresses.

## Figures and Tables

**Table 2:** Endophytic fungi species acting through induced resistance *.

Endophytic Fungi	Hosts	Pathogens Targets	References
		**Fungi**	
*Gilmaniella* sp.	*Atractylodes lancea*	-	[172]
*Phialomyces macrosporus*	*Coffea arabica*	*Colletotrichum gloeosporioides*	[189]
*Myrothecium leucotrichum*, *Stachylidium bicolor*, *Periconia hispidula* and *Brachysporiella pulchra*	*Solanum lycopersicum*	*Alternaria solani*	[190]
*Neotyphodium lolii*	*Lolium perenne*	*Alternaria alternata, Curvularia lunata* and *Fusarium avenaceum*	[191]
*Fusarium solani* sensu lato	*S. lycopersicum*	*Fusarium oxysporum* f. sp. *radicis-lycopersici, Septoria lycopersici*	[192]
*Trichoderma harzianum*	*S. lycopersicum*	*Alternaria solani, Phytophthora infestans, Botrytis cinerea*	[193,194]
*T. harzianum*, *T. asperellum*, *T. atroviride*, *T. strigosum* and *T. longibrachiatum*	*Cucumis sativus*	*Colletotrichum lagenarium*	[195]
*T. virens*	*S.lycopersicum*	*Fusarium oxysporum* f. sp. *lycopersici*	[196]
*T. harzianum*	*Capsicum annum*	*Phytophthora capsici*	[197]
*Piriformospora indica*	*Hordeum vulgare, Triticum aestivum* and *Zea mays*	*Fusarium, Blumeria graminis,*	[174,175,176,177,178]
*Piriformospora indica*	*Musa* spp.	*Fusarium oxysporum* f. sp. *cubense* (FocTR4)	[21]
		**Nematodes**	
*Piriformospora indica*	*Solanum lycopersicum*	*Meloidogyne incognita*	[198]
*Piriformospora indica*	*Glycine max*	*Heterodera glycines*	[199]
*Piriformospora indica*	*Anthurium andraeanum*	-	[200]
*Phialemonium inflatum*	*Gossypium* sp.	*Meloidogyne incognita*	[201]
*Nigrospora* sp.	*Paraserianthes falcataria*	*Meloidogyne* sp.	[202]
*Penicillium brefeldianum*	*Cucumis melo*	*Meloidogyne incognita*	[203]
*Fusarium solani* sensu lato and *Fusarium oxysporum* sensu lato	*S. lycopersicum*	*Meloidogyne incognita*	[204]
*Fusarium oxysporum* sensu lato	*Arabidopsis thaliana*	*Meloidogyne incognita*	[205,206]
*Fusarium oxysporum* sensu lato	*Musa* sp.	*Radopholus similis*	[207]
*Fusarium moniliforme*	*Oryza sativa*	*Meloidogyne graminicola*	[208]
*Pochonia chlamydosporia*	*S. lycopersicum*	*Meloidogyne javanica*	[209]
*Gaeumannomyces cylindrosporus*, *Paraphoma chrysanthemicola*, *Phialophora mustea*, *Exophiala salmonis* and *Cladosporium cladosporioides*	*Pinus tabulaeformis*	*Bursaphelenchus xylophilus*	[210]
*Trichoderma atroviride*	*Solanum lycopersicum*	*Meloidogyne javanica*	[211]
*T. harzianum*	*Glycine max*	*Pratylenchus brachyurus*	[212]
*T. harzianum*	*Solanum lycopersicum*	*Meloidogyne incognita*	[193,213]
		**Viruses**	
*Hypocrea lixii*	*Allium cepa*	Iris yellow spot virus (IYSV)	[214]
*Trichoderma harzianum* and *Metarhizium anisopliae*	*Zea mays*	Sugarcane mosaic virus (SCMV)	[215]
*T. harzianum*	*Solanum lycopersicum*	Cucumber mosaic virus (CMV)	[216]
*T. asperellum*	*Arabidopsis thaliana*	Cucumber mosaic virus (CMV)	[217]
		**Bacteria**	
*T. asperellum*	*Cucumis sativus*	*Pseudomonas syringae* pv. *lachrymans*	[218]
*T. asperellum*	*Solanum lycopersicum*	*Ralstonia solanacearum*	[219]
*T. hamatum*	*Solanum lycopersicum*	*Xanthomonas euvesicatoria*	[220]

* Possible action mechanisms are commented on in the text. “-” means that the pathogen target was not identified in the cited reference. The Bold is applied to divide different kinds of plant pathogens.

**Table 3:** Endophytic fungi with effects on abiotic stresses in plants (induced systemic tolerance).

Endophytic Fungi	Hosts	Stresses	References
*Rhizopus oryzae*	*Adiantum capillus veneris*	Heat	[227]
*Aspergillus niger*	*Helianthus annuus* and *Glycine max*	Heat	[228]
*Thermomyces* sp.	*Cucumis sativus*	Heat	[226]
*Nectria haematococca*	*Solanum lycopersicum*	Drought	[234]
*Trichoderma atroviride*	*Zea mays*	Drought	[231]
*Piriformospora indica*	*Zea mays*	Droughr	[231]
*Penicillium citrinum*, *Aureobasidium pullulans* and *Dothideomycetes* sp.	*Citrus reticulata*	Drought	[225]
*Trametes hirsuta*	*Triticum aestivum*	Metal (Pb)	[224]
*Chaetomium cupreum*	*Miscanthus sinensis*	Metal (Al)	[261]
*Phialophora mustea*	*Lycopersicon esculentum*	Metal (Cd and Zn)	[240]
*Penicillium roqueforti*	*Triticum aestivum*	Metal (Ni, Cd, Cu, Zn, and Pb)	[241]
*Paecilomyces formosus* and *Penicillium funiculosum*	*Glycine max*	Metal (Ni, Cd, and Al) and Heat	[242]
*Yarrowia lipolytica*	*Zea mays*	Salinity	[222]
*Epichloë bromicola*	*Hordeum vulgare*	Salinity	[262]
*Piriformospora indica*	*Solanum lycopersicum* and *Oryza sativa*	Salinity	[247,248]
*Piriformospora indica*	*Medicago truncatula*	Salinity	[263]
*Trichoderma longibrachiatum*	*Triticum aestivum*	Salinity	[249]
*Phoma glomerata* and *Penicillium* sp.	*Cucumis sativus*	Salinity	[230]
*Fusarium verticillioides* and *Humicola* sp.	*Glycine max*	Salinity	[223,250]
*Aspergillus flavus*	*Glycine max*	Salinity	[264]
*Fusarium oxysporum* sensu lato	*Oryza sativa*	Salinity	[265]
*Cochliobolus* sp.	*Ablemoschus esculentus*	Salinity	[266]
